# Medical and Paramedical Students' Perspectives on Artificial Intelligence in a Low‐ and Middle‐Income Country: A Cross‐Sectional Study

**DOI:** 10.1002/hsr2.71046

**Published:** 2025-08-04

**Authors:** Seyyedeh Fatemeh Mousavi Baigi, Masoumeh Sarbaz, Ali Darroudi, Khalil Kimiafar

**Affiliations:** ^1^ Department of Health Information Technology School of Paramedical and Rehabilitation Sciences Mashhad Iran; ^2^ Student Research Committee Mashhad University of Medical Sciences Mashhad Iran

**Keywords:** AI, artificial intelligence, attitude, medical student, paramedical students, perspectives

## Abstract

**Introduction:**

This study aimed to investigate the attitude of medical and paramedical students at Mashhad (Northeast of Iran) regarding artificial intelligence (AI).

**Methods:**

This cross‐sectional study was conducted from April to June 2023. A predeveloped standard questionnaire was used to collect the data. We used the proportional per‐size sampling method with email distribution containing explanations and a link to a web‐based survey.

**Results:**

A total of 208 students answered the survey (response rate: 98%). We did not find any significant difference between the AI attitude of students in the academic field (*p* = 0.505), professional interest (*p* = 0.609), and educational level (*p* = 0.128). Overall, 61.9% of medical and 67.4% of paramedical students were optimistic about the role of AI. However, a considerable proportion of students struggled to accurately define AI, reflecting gaps in conceptual familiarity rather than technical knowledge (paramedical students = 56.62% and medical students = 45.26%).

**Conclusion:**

This study explored medical and paramedical students' perceived understanding of AI and their conceptual limitations. Identifying the positive and negative attitudes of students who are the future employees of the healthcare system can help planners and policymakers in the field of AI in health design the road map of the future of AI in health in the best way.

## 1 Introduction

Artificial intelligence (AI) refers to the development of intelligent systems capable of mimicking human cognitive functions, such as learning, reasoning, and problem‐solving, through the analysis of large datasets [1]. Over the past decades, AI has evolved rapidly and has been recognized as a key component of the fourth industrial revolution [2, 3, 4, 5, 6, 7]. In healthcare, AI has shown promising applications, including improving diagnostic accuracy, optimizing treatment strategies, and enhancing patient outcomes [8, 9, 10].

Among AI‐driven technologies, decision support systems have demonstrated significant utility in clinical practice, particularly in disease prediction, personalized treatment planning, and resource allocation [11, 12]. AI‐powered deep learning models are widely used in radiology, dermatology, and pathology for automated image‐based diagnosis [13, 14, 15, 16]. Machine learning—a subset of AI—enables systems to autonomously analyze vast amounts of medical data, identify complex patterns, and generate predictive models without explicit programming [17]. AI applications are expanding beyond imaging, finding use in cardiovascular signal processing, mental health diagnostics through natural language processing, and predictive modeling for early disease detection [18, 19, 20, 21, 22]. These developments highlight AI's growing role in transforming various medical domains.

Despite these advancements, the successful implementation of AI in healthcare is influenced by multiple factors, including healthcare providers' awareness, acceptance, and preparedness [23, 24, 25, 26]. Several studies have assessed physicians' perceptions of AI. For example, a study on ophthalmologists indicated that while 89% of respondents acknowledged the concept of AI, 75% believed it should be formally integrated into clinical training [27]. Another survey in Australia and New Zealand examined healthcare professionals' attitudes toward AI‐driven robotic solutions, emphasizing that the success of AI applications relies on both professional and public perception [23]. Furthermore, a study by Martinho et al. [28] identified four main perspectives among physicians regarding AI ethics: (1) AI as a beneficial tool that enhances physicians' efficiency, (2) the necessity of strict regulations, (3) trust in private technology companies, and (4) the importance of explainability to ensure clinical acceptance. Additionally, Klumpp et al. [29] highlighted AI's diverse applications in hospital settings, ranging from data management to human‐computer interaction, underscoring the importance of tailored implementation strategies.

While AI literacy is becoming an integral part of medical education in developed countries [30, 31], its integration into medical curricula in low‐ and middle‐income countries (LMICs) remains limited. AI‐driven automation has raised concerns among students regarding job displacement, ethical challenges, and the reliability of AI systems, potentially contributing to professional uncertainty [32]. However, studies assessing healthcare students' perspectives on AI are scarce, particularly in LMICs, where AI integration faces infrastructural, educational, and policy‐related barriers [33]. In many high‐income countries, structured AI training is increasingly incorporated into medical education [34], but in developing countries like Iran, AI education remains largely absent from formal curricula [33, 35]. This gap is exacerbated by limited institutional support, scarce AI research funding, and insufficient digital literacy among healthcare students. Furthermore, the COVID‐19 pandemic has highlighted the potential of AI in streamlining healthcare processes, reducing workload, and minimizing diagnostic errors [36], making AI education more critical than ever.

Understanding students' knowledge, attitudes, and educational needs regarding AI is essential for developing AI‐integrated medical curricula tailored to the specific challenges of LMICs. Most previous studies have focused on high‐income countries, with limited evidence on how students in resource‐limited settings perceive AI and its implications for their future careers [37]. This study aims to explore the perceived understanding and attitudes of medical and paramedical students toward AI at Mashhad University of Medical Sciences (MUMS), Iran. Specifically, we seek to:

1. Evaluate students' AI literacy and perceptions across different medical and paramedical disciplines.
2. Highlight areas of conceptual uncertainty and barriers to AI education in an LMIC setting.
3. Determine preferred AI learning approaches and students' perspectives on curriculum integration.

By addressing these objectives, this study contributes to the growing discourse on AI education in healthcare, providing insights to guide curriculum development in LMICs and ensure the future workforce is adequately prepared for AI‐driven transformations in medicine.

## 2 Material and Methods

### 2.1 Study Design

The period of this cross‐sectional investigation was April–June 2023. According to Helinsky's assertion, every ethical research guideline has been followed in this investigation. The students gave their informed consent before participating in the study once it was explained to them. All personal data has been used in a discreet manner, without revealing identities.

### 2.2 Sample Size

The research population included medical and paramedical students of MUMS, Mashhad, Iran, in all available fields. Hence, the sample size of each faculty and different fields was determined independently and by the proportional per‐size method. The sample size was determined based on the Cochran formula for limited populations with 0.05, pq0.5, a tolerable error rate of *d* = 0.1, and given the total population size (2191 medical students and 913 paramedical students). Accordingly, a sample of 87 individuals was obtained for paramedical students, and 96 samples were obtained for medical students.

n = The required sample size for the research design.

z = The statistic value is equal to the standard normal curve area under curve 2.

p and q = The frequency ratio of the desired trait in the target population.

d = Effect size or precision.

$$n=\frac{\mathrm{Nqp}z^{2}}{{\mathrm{Nd}}^{2}+\mathrm{pq}z^{2}}.$$

### 2.3 Eligibility Criteria

The inclusion criteria were that all medical and paramedical students were studying at the time of this study. On the other hand, the exclusion criteria included transfer and guest students from other universities, students who were on academic leave at the time of the study, and foreign students.

### 2.4 Data Collection

In this study, a predeveloped and validated questionnaire designed by Teng et al. [18] was used. The original English version was translated into Persian using the forward and backward translation method based on WHO guidelines [38]. Two independent bilingual translators first translated the tool from English to Persian, then back‐translated it to English, and finalized the version through consensus to ensure conceptual equivalence.

The final questionnaire consisted of 15 items, including 11‐point Likert‐scale questions, multiple‐choice items, rank‐ordering questions, and one open‐ended item. The first item asked participants to provide their own definition of AI. These open‐ended responses were independently reviewed and coded by two researchers from health informatics and medical education. Definitions were categorized into four groups: (1) accurate (mentioning autonomous decision‐making, learning, or adaptability); (2) partially accurate (e.g., referring to machine learning only); (3) inaccurate; and (4) “I don't know”. Discrepancies were resolved by a third expert in clinical education. This categorization framework was adapted from Teng et al. (2022), and the standard AI definition used in the survey—“software that can learn from experience, adapt to new inputs, and make decisions”—served as the coding reference.

For the remaining items, students were instructed to refer to the standardized AI definition provided earlier in the survey. These items assessed students' attitudes and sentiments toward AI, such as their optimism, concerns, ethical awareness, and perspectives on AI's anticipated role in their careers and education. Several of these questions employed 11‐point Likert scales (0 = strongly disagree, 10 = strongly agree), enabling quantitative analysis of self‐reported perceptions. Additionally, students indicated their interest in receiving AI‐related education and their preferred formats for such training (e.g., short workshops, formal coursework, or graduate‐level programs). While these items did not directly assess technical knowledge, they offered insight into students' self‐perceived familiarity with AI and the areas in which they felt a need for further education.

The questionnaire covered six major domains: (1) demographic data (age, gender, educational level, and field of study), (2) perceived awareness of AI, (3) attitudes toward the potential impact of AI, (4) preferences for AI curriculum integration, (5) priorities in AI education design, and (6) time and setting preferences for learning AI. The final survey was developed electronically and distributed via institutional email lists among medical and paramedical students at Mashhad University of Medical Sciences.

### 2.5 Questionnaire Validation

Following the translation of the questionnaire into Persian, face validity was evaluated by five nurses and five physicians, who provided feedback on the content, clarity, readability, and simplicity. Based on their input, the final editions of the questionnaire was formulated. Subsequently, the content validity of the questionnaire was assessed using a six‐member expert panel consisting of specialists in biostatistics, health information management, medical informatics, one specialist physician, and one nurse, employing the Delphi method. The content validity ratio (CVR) and content validity index (CVI) were computed for this target. The minimum acceptable CVR values for the one‐sided tests proposed by Lawshe were 0.99 for the adequacy of content validity for six experts [39]. Following this, to calculate the validity index, experts provided their opinions on the relevance, clarity, simplicity, and transparency of each questionnaire item using a four‐part Likert scale: “completely relevant,” “relevant but requires revision,” “requires significant revision,” and “not relevant.” Consequently, the ratio of the number of experts who rated the questions as “completely relevant” and “relevant but requires revision” to the total number of experts was computed, yielding a CVI value of 0.98 for the developed tool, which is considered acceptable. Finally, to evaluate the internal reliability of the research questionnaire, the Cronbach's alpha method was utilized. Ultimately, the Cronbach's alpha coefficient for the research instrument was determined to be 0.76.

### 2.6 Statistical Analysis

SPSS VE 11 software was used for data analysis. First, in general, the condition of normality was measured by the Shapiro‐Wilk method, and if the data distribution was not normal or the data were not normalized (after changing the variable), nonparametric techniques were used; alternatively, parametric techniques were used. ANOVA test was used to compare the findings and chi‐square test was used to check the independence between qualitative variables.

## 3 Results

A total of 208 students answered the survey (response rate: 98%). In light of Table 1, the majority of paramedical students (*n* = 63; 55.8%) were in the age group of 22–19 years, and the majority of medical students (*n* = 59; 62.1%) were in the age group of 23–26 years. The majority of paramedical students were female (*n* = 74; 64.6%), and the majority of medical students were male (*n* = 49; 51.6%). Also, the majority of paramedical students were at the continuous bachelor's level (*n* = 84; 74.3%), and the majority of medical students were at the general doctorate level (*n* = 84; 88.4%). Twenty‐eight medical students (29.5%) were in the field of basic sciences, and 24 (21.2%) were in the field of health information technology.

**Table 1:** Details about the participants' demographics.

Variables	Medical students		Paramedical students	
Gender				
Female	46 (48.4)		74 (64.6)	
Male	49 (51.6)		40 (35.4)	
	95 (100.0)		113 (100.0)	
Age				
19–22	27 (28.4)		63 (55.8)	
23–26	59 (62.1)		34 (30.1)	
27–30	7 (7.4)		9 (8.0)	
31–40	1 (1.1)		7 (6.2)	
> 40	1 (1.1)		0	
Grade				
Continuous BSc	1 (1.1)		84 (74.3)	
Discontinuous BSc	1 (1.1)		5 (4.4)	
MSc	9 (9.5)		17 (15.0)	
MD	84 (88.4)		0	
Ph.D	0		7 (6.2)	
Year of entry				
2014	2 (2.2)		0	
2015	2 (2.1)		3 (2.7)	
2016	12 (12.6)		3 (2.7)	
2017	10 (10.5)		0	
2018	17 (17.9)		4 (3.5)	
2019	22 (23.2)		15 (13.3)	
2020	6 (6.3)		19 (16.8)	
2021	20 (21.1)		36 (31.9)	
2022	4 (4.2)		33 (29.2)	
Field of study	Basic science	28 (29.5)	Physiotherapy	19 (16.8)
	Intern	19 (20.0)	Occupational therapy	14 (12.4)
	Stager 1	17 (17.9)	Health information technology	24 (21.2)
	Stager 2	14 (14.7)	Social work	4 (3.5)
	Physiopath 1	4 (4.2)	Laboratory sciences	10 (8.8)
	Physiopath 2	13 (13.7)	Radiology	13 (11.5)
			Optometry	16 (14.2)
			Speech Therapy	13 (11.5)
Total	95 (100.0)		113 (100)	

Table 2 presents descriptive data about the AI attitude among the participants. In total, 88.4% of medical students and 85.8% of paramedical students with a score above 5 supported the development of AI in their field of study. Also, 94.8% of medical students and 90.4% of paramedical students with a score above five stated that AI affects their careers. The majority of medical (78.9%) and paramedical (82.3%) students stated that the principles of AI should be taught to healthcare students. Also, the majority of medical students (73.7%) and paramedical students (72.5%) stated that they are aware of the ethical ramifications of using AI in their line of work.

**Table 2:** Descriptive information regarding AI attitudes among participants.

Questions	Variables	Medical students	Paramedical students
Which of the following, if you are able to select more than one, best sums you up overall?	1. In the future, I hope to pursue a career in research.	37 (38.9)	48 (42.5)
	2. In the future, I hope to launch my own business or practice.	12 (12.6)	34 (30.1)
	3. I wish to concentrate solely on clinical work.	21 (22.1)	16 (14.2)
	4. (1,2)	13 (13.7)	9 (8)
	5. (1,3)	4 (4.2)	1(0.9)
	6. (2,3)	5 (5.3)	2 (1.8)
How much do you support or oppose the development of AI in your field of study, on a scale of 0–10? 10 is strong support; 5 is neutral; and 0 is firmly opposed.	1	1 (1.1)	1 (0.9)
	2	2 (2.1)	0
	3	5 (5.3)	3 (2.7)
	4	1 (1.1)	1 (0.9)
	5	2 (2.1)	11 (9.7)
	6	8 (8.4)	10 (8.8)
	7	11 (11.6)	14 (12.4)
	8	20 (21.1)	21 (18.6)
	9	12 (12.6)	10 (8.8)
	10	33 (34.7)	42 (37.2)
AI, in my opinion, will affect my career.	1	0	0
	2	0	0
	3	1 (1.1)	1 (0.9)
	4	1 (1.1)	2 (1.8)
	5	3 (3.2)	8 (7.1)
	6	3 (3.2)	3 (2.7)
	7	17 (17.9)	14 (12.4)
	8	8 (8.4)	28 (24.8)
	9	9 (9.5)	15 (13.3)
	10	53 (55.8)	42 (37.2)
In my opinion, AI fundamentals should be taught to healthcare students.	1	0	0
	2	0	0
	3	3 (3.2)	5 (4.4)
	4	4 (4.2)	5 (4.4)
	5	13 (13.7)	10 (8.8)
	6	11 (11.6)	8 (7.1)
	7	14 (14.7)	12 (10.6)
	8	14 (14.7)	16 (14.2)
	9	5 (5.3)	17 (15)
	10	31 (32.6)	40 (35.4)
I am aware of the moral ramifications of using AI in my line of work.	1	1 (1.1)	0
	2	3 (3.2)	1 (0.9)
	3	2 (2.1)	2 (1.8)
	4	6 (6.3)	7 (6.2)
	5	13 (13.7)	21 (18.6)
	6	8 (8.4)	10 (8.8)
	7	12 (12.6)	19 (16.8)
	8	21 (22.1)	21 (18.6)
	9	7 (7.4)	11 (9.7)
	10	22 (23.2)	21 (18.6)
The use of AI in my field gives me optimism.	1	1 (1.1)	3 (2.7)
	2	3 (3.2)	2 (1.8)
	3	4 (4.2)	3 (2.7)
	4	2 (2.1)	4 (3.5)
	5	7 (7.4)	12 (10.6)
	6	12 (12.6)	11 (9.7)
	7	16 (16.8)	7 (6.2)
	8	14 (14.7)	21 (18.6)
	9	7 (7.4)	17 (15)
	10	29 (30.5)	33 (29.2)
Regarding the part AI will play in my field, I am concerned.	1	9 (9.5)	8 (7.1)
	2	6 (6.3)	6 (5.3)
	3	14 (14.7)	6 (5.3)
	4	5 (5.3)	11 (9.7)
	5	14 (14.7)	21 (18.6)
	6	5 (5.3)	16 (14.2)
	7	6 (6.3)	8 (7.1)
	8	11 (11.6)	14 (12.4)
	9	10 (10.5)	7 (6.2)
	10	15 (15.8)	16 (14.2)
AI is a technology, in my opinion, that has to be handled carefully.	1	1 (1.1)	0
	2	0	0
	3	0	0
	4	1 (1.1)	0
	5	3 (3.2)	7 (6.2)
	6	3 (3.2)	2 (1.8)
	7	13 (13.7)	9 (8)
	8	12(12.6)	17 (15)
	9	13 (13.7)	16 (14.2)
	10	49 (51.6)	62 (54.9)
Please sum up your thoughts about AI in your field in one word or sentence:	Optimist	64 (67.4)	70 (61.9)
	Pessimistic	19 (20)	24(21.2)
	No comments	12 (12.6)	19 (16.8)
In a single sentence, define AI.	I do not know	35 (36.8)	54 (47.8)
	Correct	25 (26.3)	19 (16.8)
	Partial correct	27 (28.4)	30 (26.5)
	Incorrect	8 (8.4)	10 (8.8)
What is the timeline you see for AI to affect your career?	in 5 years	19 (20)	27 (23.9)
	in 10 years	32 (33.7)	47 (41.6)
	in 20 years	37 (38.9)	23 (20.4)
	in 50 years	6 (6.3)	13 (11.5)
	not in my lifetime	1(1.1)	3 (2.7)
Should you include instruction on the fundamentals of AI in your curriculum or should it be extracurricular and done outside of it?	Must be part of my curriculum	52 (54.7)	69 (61.1)
	Must be outside of curriculum time	41 (43.2)	42 (37.2)
Which of the following events, aimed at providing an overview of AI fundamentals, would you be interested in attending? (Able to choose more than one)	1‐day course	8 (8.4)	19 (16.8)
	Several series of workshops	57 (60)	58 (51.3)
	or 2‐h workshop	10 (10.5)	13 (11.5)
	Graduate‐level education (Master's, PhD)	18 (18.9)	23 (20.4)
		2 (2.1)	0

Questions	Variables	Participants	First priority	Second priority	Third priority	Fourth priority	Fifth priority	Sixth priority	Seventh priority
Which three goals—if your curriculum introduced the fundamentals of AI—would you prioritize?	Determine whether technology is suitable in a certain therapeutic setting.	Medical students	10 (10.5)	16 (16.8)	11 (11.6)	17 (17.9)	14 (14.7)	12 (12.6)	15 (15.8)
		Paramedical students	18 (15.9)	16 (14.2)	13 (11.5)	15 (13.3)	12 (10.6)	20 (17.7)	19 (16.8)
	Comprehend and evaluate outcomes produced by AI	Medical students	11 (11.6)	(27.4)	19(20)	14 (14.7)	8 (8.4)	7 (7.4)	10 (10.5)
		Paramedical students	11 (9.7)	17 (15)	19 (16.8)	22 (19.5)	20 (17.7)	11 (9.7)	13 (11.5)
	Possess the ability to explain technology to people in a way that they can comprehend.	Medical students	5 (5.3)	9 (9.5)	12 (12.6)	17 (17.9)	24 (25.3)	13 (13.7)	15 (15.8)
		Paramedical students	19 (16.8)	17 (15)	15 (13.3)	15 (13.3)	19 (16.8)	14 (12.4)	14 (12.4)
	Determine the moral ramifications of applying AI to medical settings.	Medical students	14 (14.7)	12 (12.6)	16 (16.8)	8 (8.4)	14 (14.7)	20 (21.1)	11 (11.6)
		Paramedical students	13 (11.5)	10 (8.8)	17 (15)	16(14.2)	13 (11.5)	22 (19.5)	22 (19.5)
	Recognize the operation of the underlying technological processes.	Medical students	13 (13.7)	7 (7.4)	10 (10.5)	12 (12.6)	17(17.9)	15 (15.8)	21 (22.1)
		Paramedical students	23 (20.4)	18 (15.9)	12 (10.6)	17(15)	17 (15)	8 (7.1)	18 (15.9)
	Acquire knowledge of the jargon to facilitate communication and teamwork with engineers and developers.	Medical students	10 (10.5)	9	(9.5)	15 (15.8)	13 (13.7)	19 (20)	19 (20)
		Paramedical students	7 (6.2)	15 (13.3)	15 (13.3)	12 (10.6)	20 (17.7)	24 (21.2)	20 (17.7)
	Determine how AI may enhance the quality of healthcare.	Medical students	32 (33.7)	15 (15.8)	18 (18.9)	12 (12.6)	5 (5.3)	9 (9.5)	4 (4.2)
		Paramedical students	22 (19.5)	20 (17.7)	22 (19.5)	16 (14.2)	12 (10.6)	14 (12.4)	7 (6.2)

Furthermore, 82% of medical students and 78.7% of paramedical students stated that using AI in my field gives me optimism. However, 49.5% of medical students and 54.1% of paramedic students stated that they are concerned about the role AI will play in their field. In this regard, students (medicine: 46.4%; paramedicine: 93.9%) stated that AI is a technology that should be used carefully.

When asked to comment on their sentiments toward AI, medical and paramedical students were largely upbeat about the role of AI (paramedical students = 61.9%; medical students = 67.4%). Also, when students were asked to describe AI in one sentence, 56.62% of paramedic students and 45.26% of medical students defined it incorrectly or stated that they did not know.

Students were questioned: Should AI principles be taught outside of the classroom or as part of the curriculum? The majority of them answered (medical students: 61.1%; paramedical students: 54.7%) that it should be part of my curriculum or educational program. Additionally, most medical and paramedical students predicted that within the next ten to twenty years, AI would be included in their field.

On the other hand, when students from various disciplines were asked which three goals were most important when introducing AI principles into their curricula, most medical students consistently ranked the following among the top goals: (1) identify ways in which AI can improve the quality of healthcare; (2) understand and interpret results generated by AI. (3) Identify the ethical implications of using AI in clinical disciplines. On the other hand, most of the paramedical students' priorities were as follows: (1) Understand how basic technological processes work. (2) Identify ways in which AI can boost the quality of healthcare. (3) Understand and interpret the results generated by AI.

**Figure 1:**
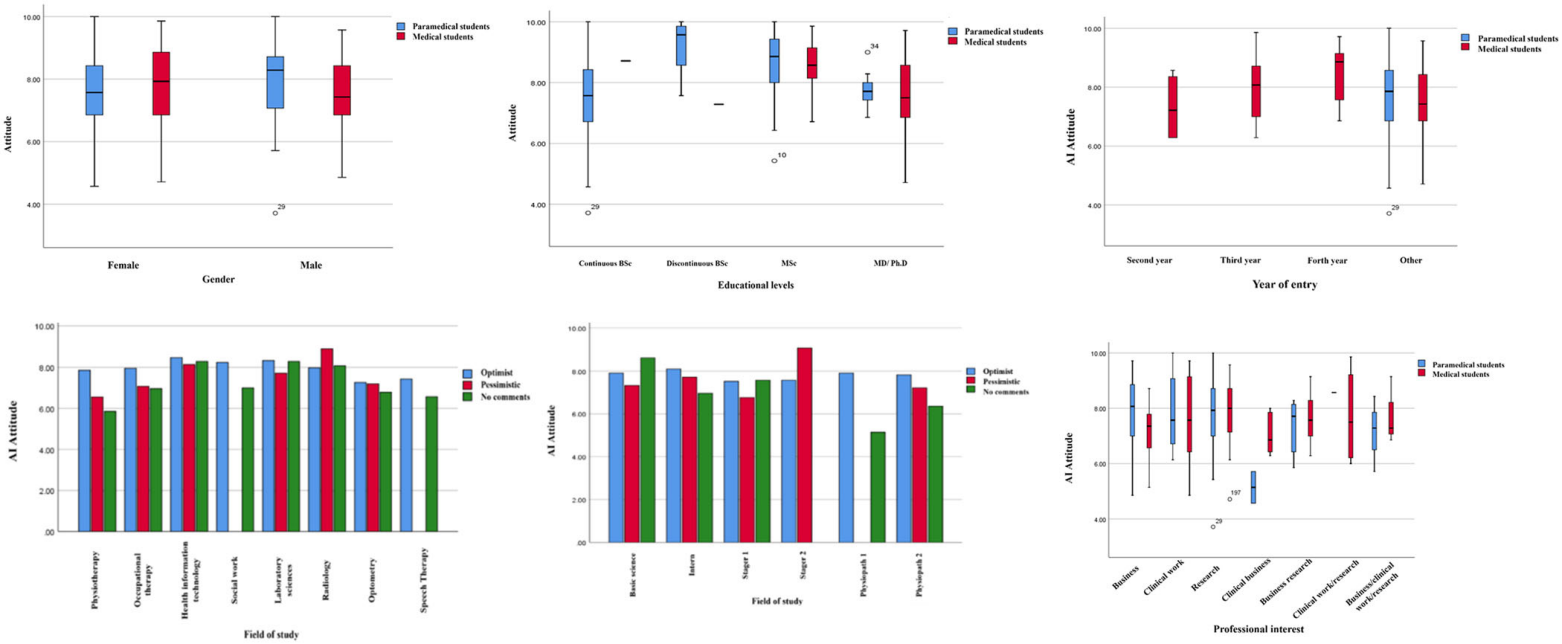
Attitude towards AI classified by gender, educational levels, year of entry, fields of study, and professional interests.

To examine differences in students' attitudes toward AI based on variables such as gender, academic level, year of entry, field of study, and professional interest, a one‐way analysis of variance (ANOVA) was conducted. The results indicated no significant differences in students' AI attitudes across these variables. The effect size (η²) and 95% confidence intervals (CI) for each variable were as follows: gender (*F*(1, 206) = 1.53, *p* = 0.217, *η*² = 0.014, 95% CI [−0.22, 0.09]), academic level (*F*(3, 204) = 2.10, *p* = 0.128, *η*² = 0.018, 95% CI [−0.20, 0.11]), year of entry (*F*(9, 198) = 0.89, *p* = 0.473, *η*² = 0.005, 95% CI [−0.16, 0.12]), field of study (*F*(5, 202) = 0.82, *p* = 0.505, *η*² = 0.007, 95% CI [−0.18, 0.15]), and professional interest (*F*(6, 201) = 0.64, *p* = 0.609, *η*² = 0.003, 95% CI [−0.14, 0.10]). These findings suggest that none of these demographic and academic factors had a notable impact on students’ attitudes toward AI (Figure 1).

**Figure 2:**
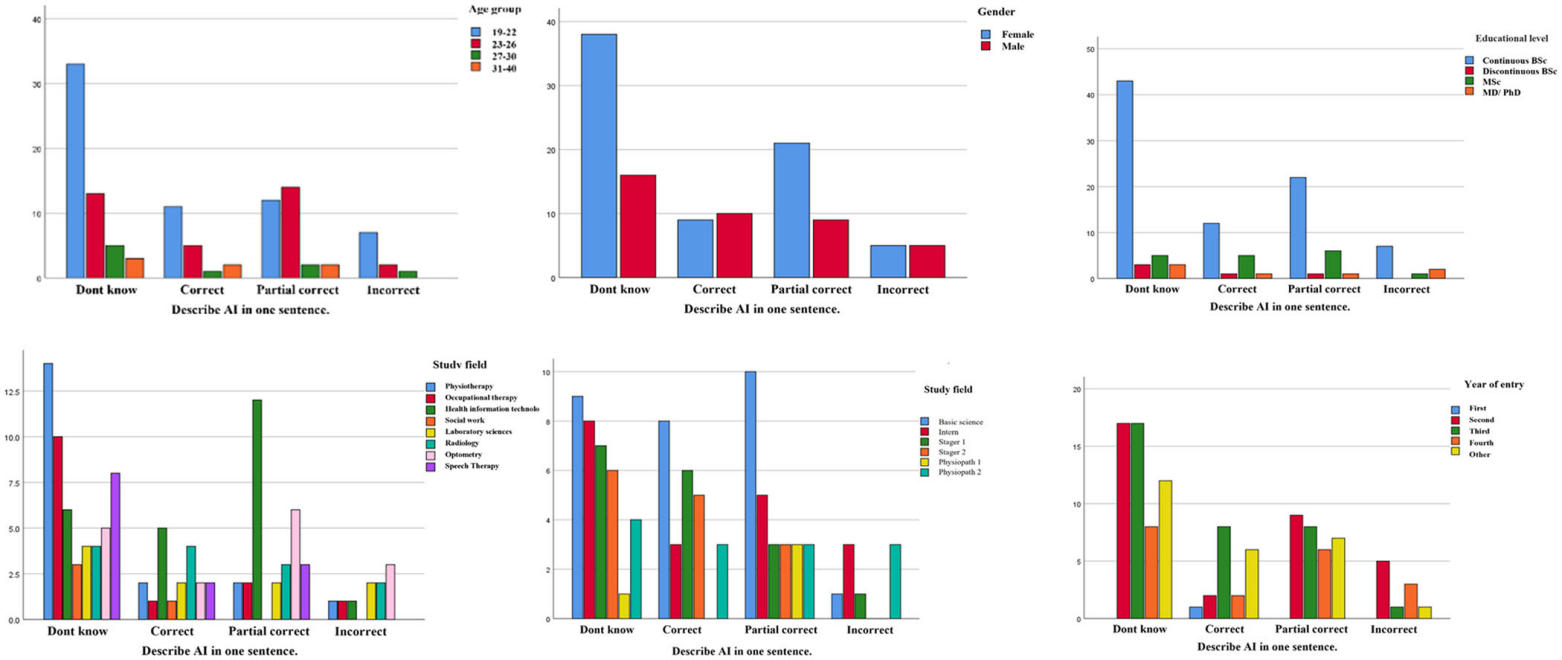
Knowledge towards AI classified by age groups, gender, educational levels, year of entry, and fields of study.

Furthermore, to assess differences in students' knowledge of AI based on age group, academic level, year of entry, gender, and field of study, a Chi‐Square test was performed. The results indicated no significant associations between these variables and students' AI knowledge. The effect size (Cramer's V) and 95% confidence intervals for each variable were as follows: age group (χ²(9, *N* = 208) = 7.45, *p* = 0.590, Cramer's *V* = 0.08, 95% CI [0.03, 0.14]), gender (*χ*²(3, *N* = 208) = 4.57, *p* = 0.206, Cramer's *V* = 0.10, 95% CI [0.04, 0.15]), academic level (*χ*²(9, *N* = 208) = 8.26, *p* = 0.508, Cramer's *V* = 0.07, 95% CI [0.02, 0.12]), year of entry (χ²(12, *N* = 208) = 14.35, *p* = 0.279, Cramer's *V* = 0.09, 95% CI [0.03, 0.14]), and field of study (χ²(21, *N* = 208) = 29.14, *p* = 0.111, Cramer's *V* = 0.11, 95% CI [0.05, 0.16]). These findings indicate that students' knowledge of AI did not significantly differ across demographic and academic categories (Figure 2).

## 4 Discussion

### 4.1 Principal Findings

This study found that many medical and paramedical students demonstrated limited conceptual clarity and self‐reported familiarity with AI, particularly in defining core concepts. These findings underscore the urgent need for incorporating structured AI education within medical and paramedical curricula. As AI continues to be integrated into healthcare systems, equipping future professionals with foundational knowledge and awareness is essential. Despite these limitations, most students expressed optimistic views regarding AI's role in their future careers (paramedical students: 61.9%; medical students: 67.4%).

No significant differences were observed in attitudes toward AI across different educational levels (*p* = 0.128) or among students with varying professional interests (*p* = 0.609). This suggests a broad acceptance of AI among students from diverse academic backgrounds. Our findings align with previous international studies. For instance, a multicenter study in Germany reported that medical students did not express significant concern about AI replacing radiologists [40]. Similarly, a study conducted in Pakistan found that medical students held favorable attitudes toward AI [5]. In the United Kingdom, a survey of general practitioners indicated that AI could enhance physician efficiency, although ethical concerns were also raised [41]. Likewise, a global survey of pathologists revealed a predominantly positive attitude toward AI as a tool for improving efficiency and quality assurance, whereas a separate study in South Korea suggested that physicians there do not anticipate AI replacing their roles [42].

In our study, 56.62% of paramedical students and 45.26% of medical students were unable to accurately define AI or admitted to lacking knowledge about it. This finding is consistent with an online survey conducted in southern Vietnam, where 92.2% of respondents reported having no specific knowledge of AI. However, 77.9% believed AI would be beneficial for their careers, and 77.2% expected it to play a role in public health monitoring and epidemic prevention [43]. Addressing this knowledge gap, the Ontario Medical Students Association has advocated for the integration of AI education into medical curricula [44]. A lack of understanding regarding AI integration in healthcare and misinterpretation of AI‐generated results could lead to distrust and hesitation in adopting these technologies. Our findings suggest that incorporating AI education into medical training can alleviate concerns and facilitate the acceptance of AI in the healthcare community. Similar findings were reported in a study of medical physicists and oncologists in New Zealand, which demonstrated a positive correlation between prior AI exposure and its acceptance [45]. However, to effectively integrate AI into healthcare systems—especially in LMICs—educational initiatives must be accompanied by structural reforms in data and information infrastructures. Recent reviews in the field of health informatics highlight the fragmentation of registration systems and the lack of national strategies for interoperable digital platforms. These limitations restrict the broader implementation of intelligent technologies, underscoring that improving students' AI literacy must be paralleled by the development of cohesive, technology‐ready ecosystems in healthcare [46].

A systematic review by Mousavi Baigi et al., analyzing 38 studies, found that although students generally held positive attitudes toward AI, most had limited exposure to its practical applications and lacked hands‐on experience [33]. Additionally, a more recent review examining AI literacy among healthcare professionals and students reported that in nearly half of the included studies, participants demonstrated very low levels of self‐reported AI knowledge and confidence in applying AI tools [47]. These findings highlight the need for structured and accessible AI education to improve students' familiarity with core concepts and their readiness for AI integration. Supporting this conclusion, Sun and Medaglia [48] and Ghaddaripouri [49] emphasized that insufficient awareness and understanding are among the key perceived barriers to the successful adoption of AI in healthcare settings.

Conversely, Chen et al. found that while medical students and professionals recognize the growing role of AI in clinical practice, they lack hands‐on experience and practical knowledge. Their study, which assessed AI acceptance among medical professionals worldwide, concluded that although participants had a generally positive attitude toward AI, they remained cautious. The study also recommended that increased AI education could help reduce concerns and resistance to its adoption [50].

Regarding ethical considerations, 73.7% of medical students and 72.5% of paramedical students (scoring above 5) indicated an understanding of AI's ethical implications. AI introduces new ethical challenges, such as increased bias risks and the potential for diagnostic errors, which may result in over‐ or under‐diagnosis with significant clinical consequences [51]. As a result, medical ethics education must evolve in parallel with the anticipated acceleration of AI development in medicine [52]. However, AI ethics education in medical programs remains insufficient. A study involving 487 medical students from Germany, Austria, and Switzerland revealed that most had received little to no formal training on AI ethics [53, 54].

In our study, concerns regarding AI's impact on students' future careers were apparent. Many students predicted that AI would become an integral part of their profession within the next 10–20 years. In this regard, research conducted by Deloitte [55] and in collaboration with the Oxford Martin Institute [56] suggested that AI could automate 35% of UK jobs within the same time frame. Other studies have highlighted that occupations involving repetitive and predictable tasks are most susceptible to automation [57, 58]. However, some studies argue that AI could enhance job quality and improve wealth distribution [59]. It is increasingly evident that AI will not entirely replace medical professionals but will serve as an auxiliary tool to enhance patient care [52].

### 4.2 Strengths and Limitations

This study has several limitations. First, self‐selection bias may have influenced participant recruitment and engagement. While this study did not aim to comprehensively assess technical knowledge of AI, the open‐ended definition item served as a proxy to explore students' conceptual clarity, whereas the remaining items focused on perceptions, attitudes, and educational preferences. Second, the study focused exclusively on the attitudes and knowledge of medical and paramedical students, leaving out the perspectives of practicing clinicians and specialists. Third, the study was conducted at a single institution, Mashhad University of Medical Sciences (MUMS) in Iran. However, considering that Mashhad is Iran's second most populous city and that MUMS is one of the country's leading medical universities, the findings may still be generalizable to other middle‐ and low‐income countries.

One of the study's key strengths is the use of a standardized questionnaire, whose validity had already been established. Additionally, this study compared the perspectives of students from various medical and paramedical disciplines, providing a comprehensive understanding of their views on AI. Unlike many previous studies that primarily focused on high‐income countries, this study offers insights into a middle‐income setting. Future studies could further explore patient perspectives on AI in healthcare. Additionally, qualitative interviews with key stakeholders could be conducted to assess their readiness for integrating AI into clinical and rehabilitative practices.

## 5 Conclusion

This study assessed students' perceived understanding of AI and revealed areas of conceptual ambiguity based on their ability to define AI and their self‐reported familiarity. Although most students expressed optimism about the integration of AI in their future careers, the findings suggest that many lacked foundational conceptual clarity. Importantly, students consistently reported diverse educational needs regarding AI, with the majority supporting its inclusion in the formal curriculum. To effectively prepare the future healthcare workforce for AI‐driven transformations, targeted curriculum development should be informed by students' attitudes, perceived readiness, and preferences. Recognizing these dimensions can guide policymakers and educators in designing responsible, context‐appropriate AI literacy programs.

## Author Contributions

**Seyyedeh Fatemeh Mousavi Baigi:** conceptualization, writing – original draft, writing – review and editing, methodology, formal analysis, resources. **Masoumeh Sarbaz:** validation, writing – review and editing, formal analysis. **Ali Darroudi:** formal analysis, data curation, writing – review and editing. **Khalil Kimiafar:** writing – review and editing, conceptualization, project administration, supervision.

## Ethics Statement

The ethics committee of Mashhad University of Medical Sciences granted approval for this study (approval number: IR.MUMS.FHMPM.REC.1403.242).

## Conflicts of Interest

The authors declare no conflicts of interest.

## Transparency Statement

The lead author Khalil Kimiafar affirms that this manuscript is an honest, accurate, and transparent account of the study being reported; that no important aspects of the study have been omitted; and that any discrepancies from the study as planned (and, if relevant, registered) have been explained.

## Data Availability

The data that support the findings of this study are available from the corresponding author upon reasonable request.
